# Warfarin-Induced Pulmonary Hemorrhage: A Rare and Life-Threatening Complication in the Resource-Limited Setting of Gaza

**DOI:** 10.1155/crpu/8866411

**Published:** 2025-08-17

**Authors:** Abdallah Abu Shammala, Majed Jaber, Bilal Irfan, Alaa Owda, Abdallah Herzallah

**Affiliations:** ^1^Department of Internal Medicine, European Gaza Hospital, Khan Yunis, Gaza Strip, State of Palestine; ^2^Department of Neurology, University of Michigan Medical School, Ann Arbor, Michigan, USA; ^3^Center for Bioethics, Harvard Medical School, Boston, Massachusetts, USA; ^4^Center for Surgery and Public Health, Brigham & Women's Hospital, Boston, Massachusetts, USA; ^5^Department of Epidemiology, University of Michigan School of Public Health, Ann Arbor, Michigan, USA

## Abstract

Warfarin is widely prescribed for the prevention of thromboembolic events but carries a well-known risk of bleeding complications. While gastrointestinal and intracranial hemorrhages remain among the more frequent sites, pulmonary hemorrhage is an exceedingly rare case presentation and can be particularly catastrophic. We report the case of a 57-year-old male in Gaza with an extensive medical history of past hospitalization, who presented with severe ecchymosis, with his condition progressing to life-threatening pulmonary hemorrhage. This case occurred amid an active Israeli military assault in November 2024 in the European Gaza Hospital in Khan Yunis, at a time when diagnostic modalities had become limited or completely destroyed and healthcare resources were in severe shortage. The patient was treated with supportive measures, including mechanical ventilation and warfarin reversal using vitamin K and fresh frozen plasma. His anticoagulation regimen was changed to rivaroxaban. This case underlines the difficulties in diagnosing and managing rare but critical hemorrhagic complications in conflict-affected regions and emphasizes the urgency for clinicians to be vigilantly monitoring international normalized ratio values to conduct timely interventions.

## 1. Introduction

Warfarin has served as an important anticoagulant for decades, used in conditions such as atrial fibrillation, venous thromboembolism, and mechanical heart valves [1]. By inhibiting vitamin K-dependent clotting factors, warfarin confers substantial protection against thrombotic events, yet it has been noted to be notoriously difficult to manage due to the interaction of various drugs, dietary influences, genetic variations, and its narrow therapeutic window [2]. This renders it challenging to maintain a therapeutic international normalized ratio (INR). Supratherapeutic INR, defined as an INR significantly above the therapeutic range, predisposes patients to an increased risk of bleeding. Among the most commonly reported sites of warfarin-associated bleeds are those occurring in the gastrointestinal or intracranial compartments, thus rendering pulmonary hemorrhage as extraordinarily rare manifestation [3]. It has been overlooked to some degree despite its potential severity as a warfarin-induced coagulopathy.

Pulmonary hemorrhage broadly refers to bleeding within the alveoli or lung parenchyma and is typically associated with autoimmune, such as systemic lupus erythematosus and Goodpasture syndrome, infectious, or traumatic etiologies [4]. Drug-induced pulmonary hemorrhage has been described in a few case reports, with warfarin-associated bleeding accounts representing a small fraction of these [5]. Timely recognition of such a potentially fatal complication is crucial, especially in low-resource or conflict settings. This case presented in November 2024 at the European Gaza Hospital (EGH) in Khan Yunis, located in a conflict-ridden and resource-limited environment, illustrates the compounded difficulties in diagnosing and managing warfarin-induced pulmonary hemorrhage during an active Israeli military assault.

## 2. Case Presentation

A 57-year-old male with a history of atrial fibrillation, Type 2 diabetes mellitus, hypertension, heart failure, chronic kidney disease (CKD), and an old cerebrovascular accident (CVA), with residual right-sided weakness, presented to our emergency department at EGH with extensive bruising over the right arm, shoulder, chest, and abdomen. He indicated he did not experience epistaxis, hemoptysis, hematemesis, melena, sudden losses of consciousness, or any recent traumatic events. He had no history of recent hospitalization or indication of frequent bleeding episodes. His medications included metformin, gliclazide, enalapril, bisoprolol, furosemide, and warfarin. Notably, the patient had mistakenly taken warfarin twice daily, for a total of 10 mg, instead of the prescribed once-daily dose of 5 mg, for 1 month, drawn from two different generic brands.

**Figure 1:**
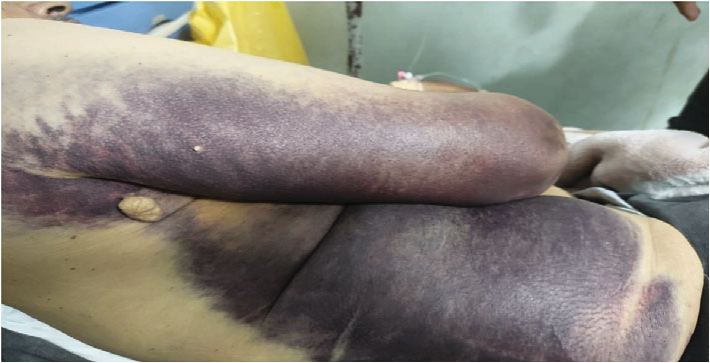
Extensive, nonpalpable ecchymosis extending from the right arm to the right chest and abdomen.

On admission, his vitals were stable (temperature 37.3°C, respiratory rate 18/min, oxygen saturation 98% on room air, blood pressure 120/70 mmHg, and heart rate 100 bpm). Physical examination revealed extensive, nonpalpable ecchymosis extending from the right arm to the right chest and abdomen, plus swelling around the right shoulder joint (Figure 1). There were no abnormal respiratory findings initially, and neurological evaluation showed his known right-sided weakness (3/5), hypertonia, and hyperreflexia but with sensations still being reported. Because he did not display any respiratory symptoms and auscultation was not notable at the time of admission, an entry CXR was inadvertently deferred, a common compromise in the Gaza setting where radiography slots are triaged toward the most symptomatic patients during mass-casualty surges.

**Figure 2:**
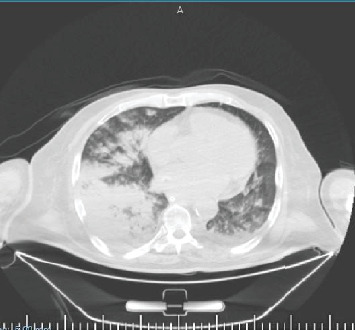
Noncontrast chest CT demonstrating consolidation in the right middle and lower lobes, consistent with pulmonary hemorrhage, and minimal bilateral pleural effusion.

Laboratory work demonstrated a hemoglobin (Hb) level of 7.1 g/dL, down from a baseline of 13 g/dL 2 weeks prior, and an INR that was noncoagulable (> 10). Serum potassium was low at 2.7 mmol/L. A focused search for bleeding included a digital rectal exam to rule out gastrointestinal bleeding, noncontrast brain CT to scan for intracranial hemorrhage, and ultrasounds of the right shoulder, lungs, and abdomen to rule out intra-articular bleeding, hemothorax, and intra-abdominal and retroperitoneal hemorrhage; no obvious source besides the large ecchymoses was identified (Figure 2). No free fluid or organized hematoma was noted. A contrast abdominal pelvic CT was not easily accessible in the given circumstances so we relied on serial abdominal examinations and CBC monitoring. The abdomen remained soft and nondistended throughout, further pointing against occult intra-abdominal or retroperitoneal hemorrhage. A urine analysis was also conducted with no hematuria presenting.

Warfarin was immediately held, and reversal therapy was initiated with vitamin K (10 mg) and six units of FFP. The patient was admitted for close monitoring of his hemoglobin and INR (Table 1). Despite these interventions, hemoglobin continued to drop, reaching 6.2 g/dL on Day 2, and INR decreased to 1.92. He received a unit of packed red blood cells (PRBCs). Over subsequent days, he required additional PRBC or FFP transfusions, guided by INR and hemoglobin trends. On Day 5, he acutely developed dyspnea and hypoxia, with a new right-sided diffuse opacity on chest X-ray. His condition deteriorated further, becoming hypotensive (90/40 mmHg) and drowsy, necessitating emergent intubation and transfer to the ICU. A chest CT showed consolidation in the right middle and lower lobes, consistent with pulmonary hemorrhage and minimal pleural effusion. Although the patient had never been recorded to have hemoptysis before this crisis, a backflow blood was noted in the ETT shortly after intubation, further corroborating a possible intrapulmonary source.

His INR had spiked back to 5.23. After additional vitamin K, FFP, and mechanical ventilation (MV), his respiratory status stabilized. He was extubated on Day 7, then transitioned to rivaroxaban on Day 8. By Day 11, with a stable hemoglobin and near-normal INR (1.23), he was safely discharged.

## 3. Discussion and Conclusions

Pulmonary hemorrhage is an atypical yet grave complication of warfarin therapy, occurring less frequently than gastrointestinal, intracranial, or urinary bleeding events [3, 6]. Historically, it has been described in select case reports, with Brown et al. highlighting an example as early as 1965 [7]. Our patient's case underscores the lethal potential of warfarin-associated pulmonary bleeding, heightened by the presence of multiple comorbidities (i.e., CKD, heart failure, and a prior stroke) that can further predispose to hemorrhagic complications [8]. Several pathophysiologic factors can precipitate pulmonary hemorrhage in patients taking warfarin. Warfarin-induced coagulopathy, once the INR exceeds therapeutic limits, can cause diffuse alveolar or parenchymal bleeding even in the absence of overt trauma [2, 9]. The patient's significant miscalculation in warfarin dosing, exacerbated by using two different generic formulations, led to extremely supratherapeutic INR values (> 10). While warfarin toxicity classically presents with easy bruising or bleeding from mucosal sites, substantial alveolar bleeding can be masked until respiratory compromise occurs, making the diagnosis challenging [10, 11].

Conducting a comprehensive evaluation of respiratory emergencies often relies on advanced imaging (e.g., high-resolution CT) and bronchoscopy with bronchoalveolar lavage (BAL) [12]. In many settings, bronchoscopy serves as the gold standard to confirm alveolar hemorrhage by demonstrating progressively bloodier aliquots and hemosiderin-laden macrophages [13]. However, in Gaza, especially amid the ongoing Israeli military assault, access to specialized diagnostic modalities was limited. Our patient's condition demanded an urgent, clinical-based approach, supported by chest imaging compatible with hemorrhage and rapid correction of coagulopathy without the benefit of bronchoscopy for definitive confirmation.

The presence of continuing military strikes complicates healthcare delivery in Gaza, restricting the procurement of essential medical supplies and limiting staffing capacities. Conflict-affected regions often struggle with supply chain disruptions, inadequate blood product availability, and compromised health infrastructure, which is particularly evident in Gaza [14–16]. Furthermore, structural damage to hospital facilities during assaults can limit the functionality of critical diagnostic and therapeutic services (e.g., advanced imaging or ICU-based procedures), placing both patients and healthcare workers at elevated risk.

This patient's story also underscores the synergy of comorbidities, especially CKD and heart failure, in amplifying bleeding risks from anticoagulants [2]. CKD can alter warfarin metabolism, while heart failure further compromises tissue perfusion, possibly shifting warfarin pharmacokinetics and exacerbating bleeding tendencies [4]. Patient education is paramount: thorough counseling on dose, frequency, and brand consistency of warfarin can avert critical dosing mishaps [2, 17]. Indeed, our patient's deadly complication might have been avoided with clearer instructions and oversight.

Immediate management of suspected warfarin-induced pulmonary hemorrhage includes urgent reversal of the coagulopathy, typically via vitamin K, prothrombin complex concentrate (PCC), or FFP [10]. In severe cases, supportive therapy, ranging from MV to extracorporeal membrane oxygenation (ECMO), may be necessary if hypoxia is profound [18]. A multidisciplinary approach is crucial, bringing together internal medicine, pulmonology (if available), critical care, and, in resource-rich settings, interventional radiology teams. Where feasible, newer direct oral anticoagulants (DOACs) with more predictable pharmacokinetics and fewer drug interactions may be an alternative for at-risk warfarin patients [19].

Ongoing hostilities in Gaza remain a critical driver of resource scarcity and pose a difficulty in even acquiring data to assess the full needs of the population and individual patients [20–22]. For patients requiring long-term anticoagulation in this conflict-ridden context, strict INR surveillance, telemedicine consultations (if feasible), and meticulously orchestrated warfarin education programs could help mitigate life-threatening complications [16]. Implementing standardized anticoagulation protocols, such as using dedicated anticoagulation clinics and smartphone-based INR self-monitoring devices, might offer partial solutions even in resource-constrained contexts [23]. However, these measures hinge upon stable infrastructure, collaborative partnerships, and the cessation of hostilities to meaningfully improve patient outcomes [24, 25].

Overall, this case not only amplifies the need for clinicians worldwide to remain vigilant about atypical sites of warfarin-induced bleeding but also calls attention to the dire logistical difficulties faced by healthcare providers in conflict zones. The prompt reversal of coagulopathy and supportive care can be life-saving, but a broad, system-wide improvement in monitoring and patient education is indispensable for preventing ill outcomes.

This case vividly demonstrates the rare yet potentially fatal nature of pulmonary hemorrhage in the setting of warfarin-induced coagulopathy. Diagnosing and managing such complications become even more daunting in a resource-limited, conflict-affected environment like Gaza. Early recognition, rapid correction of anticoagulation, and multidisciplinary supportive care were essential in saving this patient's life. Stricter warfarin dosing oversight, more regular INR assessments, and consideration of DOACs when feasible may further help reduce the incidence of warfarin-related pulmonary hemorrhage.

In Gaza, where ongoing conflict disrupts healthcare infrastructure, the challenges of managing complex cases like this are amplified. Limited access to advanced diagnostic tools, scarcity of blood products, and frequent interruptions in supply chains necessitates a heightened reliance on clinical acumen and an adaptive environment for treatment protocols. Strengthening local healthcare capacity through targeted training on anticoagulation management and bleeding complications, alongside robust patient education programs about medication adherence may be means to making some positive change. Furthermore, implementing community-based strategies for regular INR monitoring, when possible, and safe warfarin dosing could mitigate risks. This case reflects that the unique obstacles to care in Gaza may hinder life-saving measures.

## Figures and Tables

**Table 1:** Laboratory results by day and follow-up.

**Date**	**Hemoglobin (g/dL)**	**White blood cell count (per *μ*L)**	**Platelet count (per *μ*L)**	**International normalized ratio**	**Actions conducted**
Day 1 (presentation)	7.1	8.2	200	Not coagulable (>10)	FFP and vitamin K
Day 2	6.2	7.3	150	1.92	A PRBC unit
Posttransfusion	7.9	6.7	170	NA	Follow-up
Day 3	6.9	6.3	250	1.47	A PRBC unit
Day 4	8	5	210	1.89	Follow-up
Day 5	9	19	264	5.23	Vitamin K, FFP, MV
Day 6	9.5	8.8	130	3.8	Vitamin K, FFP, MV
Day 7	8.6	10.2	170	3.17	Symptoms resolved, extubated
Day 8	8	8.4	180	2.96	Transitioned to rivaroxaban
Day 9	9	6	290	1.37	Follow-up
Day 10	9	6.7	240	N/A	Follow-up
Day 11	9.5	7.4	180	1.23	Discharge

*Note:* Hemoglobin (g/dL): reference range: 13.5–17.5 (men) and 12.0–15.5 (women); white blood cell count (per *μ*L): reference range: 4000–11,000; platelet count (per *μ*L): reference range: 150,000–450,000; and INR: normal range: 0.8–1.2 (nonanticoagulated).

Abbreviations: FFP, fresh frozen plasma; MV, mechanical ventilation; PRBC, packed red blood cells.

## Data Availability

There were no datasets generated.

## Nomenclature

INR: international normalized ratio
FFP: fresh frozen plasma
PT: prothrombin time
PRBC: packed red blood cell
MV: mechanical ventilation
PCC: prothrombin complex concentrate
BAL: bronchoalveolar lavage
ECMO: extracorporeal membrane oxygenation
CT: computed tomography
ICU: intensive care unit
N/A: not available
